# Hemicrania continua associated with an unruptured anterior communicating artery aneurysm: first case report

**DOI:** 10.1186/s10194-021-01219-5

**Published:** 2021-03-08

**Authors:** Ibrahim Imam

**Affiliations:** Royal Devon and Exeter Hospital, Exeter, UK

The Editor.

Journal of Headache and Pain.

Dear Sir.

I present this 51-year-old woman who presented with a 24-month history of a gradual onset right sided headache which was continuous from the start. It was predominantly over the parieto-occipital area, the forehead and cheek, and she also had occasional sharp shooting pains over the left side of her head and neck. She had right sided photophobia but did not have nausea. The headache was associated with watering of the right eye, and occasionally blockage of the right nostril. She had not noticed any reddening of the eye. She described the headaches as disabling.

She has a history of hypertension and was on Ramipril; a temporary withdrawal did not resolve the headache. She has no relevant family history. She smokes 15 cigarettes a day but drinks alcohol only occasionally. She reported significant personal and family stress related to recent bereavement from the loss of her mother.

On examination, her right conjunctiva was slightly red. Her cranial nerves and systemic neurological examination were otherwise normal.

The clinical diagnosis of hemicrania continua was made and her headaches resolved promptly on Indomethacin but only at a dose of 50 mg three times a day. Her magnetic resonance imaging brain scan showed an 8 × 7 × 9 mm anterior communicating artery aneurysm which was confirmed with a subsequent computed tomography angiogram (Fig. 1). Unfortunately, an attempt to treat the aneurysm interventionally was unsuccessful, and she remains on Indomethacin for control of her headache.

**Fig. 1:**
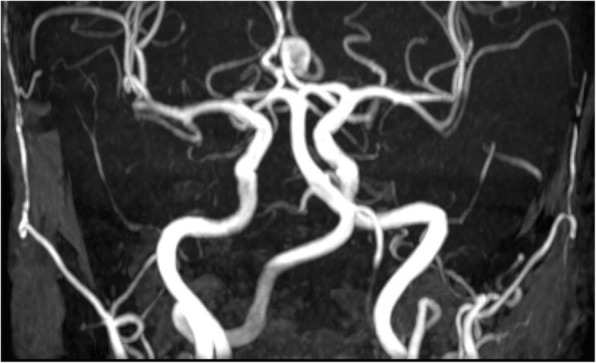
Computed tomography angiogram (CTA) of the patient demonstrating the anterior communicating artery anuerysm

## Discussion

This is the first case report of hemicrania continua in association with an anterior communicating artery aneurysm. The patient’s history and response to indomethacin are entirely consistent with hemicrania continua. Hemicrania continua predominantly affects females and it is typically a moderately severe unilateral continuous headache which may be associated with intermittent jabs and jolts as is the case with this patient [1, 2].

Hemicrania continua has been reported in association with a variety of intracranial pathologies especially traumatic brain injury, sinusitis, primary and secondary brain tumours, internal carotid artery dissection, dental, orbital and temporomandibular joint problems [3, 4]. Rarer secondary causes of hemicrania continua include carotid cavernous fistula and cerebral vein thrombosis (CVT) [5, 6]. There is however only one case report of hemicrania continua in relation to an intracranial aneurysm, and this was of the internal carotid artery [7]. Cerebral aneurysms on the other hand have been reported in association with other trigeminal autonomic cephalalgias such as cluster headaches and short-lasting unilateral neuralgiform headaches with conjunctival injection and tearing (SUNCT) [8, 9].

There is a possibility that the aneurysm in this case is coincidental, unruptured aneurysms being present in 2% of the general population [10]. In this case, it was difficult to be absolutely certain of the causative relationship of the aneurysm to her headache because the attempt to treat the aneurysm was unsuccessful. This case report nevertheless emphasises the importance of investigating patients with hemicrania continua for associated intracranial causes.

Yours sincerely.

Dr. Ibrahim Imam, FRCP.

Consultant Neurologist.

Neurology Department.

Royal Devon and Exeter Hospital.

Exeter.

EX1 5DH.

United Kingdom.
